# Supplementary material for: Health Risks and Consequences of a COVID-19 Infection for People with Disabilities: Scoping Review and Descriptive Thematic Analysis
Source: Int J Environ Res Public Health. 2021 Apr 20;18(8):4348. doi: 10.3390/ijerph18084348 (PMC8074171; doi:10.3390/ijerph18084348)
Supplement: Supplementary file 1 [file ijerph-18-04348-s001.zip › SM S1 - search terms.pdf]

Supplementary material S1

Search strategies

PubMed/Medline

("Refugees"[Mesh] OR "Refugee Camps"[Mesh] OR refugee*[tw] OR asylum[tw] OR "Human Rights"[Mesh] OR "Social Justice"[Mesh] OR justice[tw] OR injustice[tw] OR "Community Integration"[Mesh] OR "Transients and Migrants"[Mesh] OR "Emigrants and Immigrants"[Mesh] OR *migrant*[tw] OR "Foreign Professional Personnel"[Mesh] OR "Ethnic Groups"[Mesh] OR "Indigenous Peoples"[Mesh] OR "Sexual and Gender Minorities"[Mesh] OR "Gender-Based Violence"[Mesh] OR "Sexism"[Mesh] OR "Gender Identity"[Mesh] OR "Religion"[Mesh] OR "Vulnerable Populations"[Mesh] OR vulnerab*[tw] OR disadvantaged[tw] OR "Prisoners"[Mesh] OR "Enslaved Persons"[Mesh] OR "Homebound Persons"[Mesh] OR "Homeless Persons"[Mesh] OR homeless*[tw] OR "Institutionalization"[Mesh] OR "Adolescent, Institutionalized"[Mesh] OR "Child, Institutionalized"[Mesh] OR "Long-Term Care"[Mesh] OR "Crime Victims"[Mesh] OR "Disaster Victims"[Mesh] OR "Medically Uninsured"[Mesh] OR "Medically Underserved Area"[Mesh] OR underserved[tw] OR "Minority Health"[Mesh] OR "Health Equity"[Mesh] OR "Health Services Accessibility"[Mesh] OR "Poverty"[Mesh] OR "Working Poor"[Mesh] OR "Employment"[Mesh] OR "Developing Countries"[Mesh] OR "low income"[tw] OR "Race Factors"[Mesh] OR "American Native Continental Ancestry Group"[Mesh] OR "Frail Elderly"[Mesh] OR "Comorbidity"[Mesh] OR "Rural Population"[Mesh] OR "Rural Health Services"[Mesh] OR "Rural Health"[Mesh]) **AND** ("COVID-19"[tw] OR "SARS-Cov-2"[tw] OR

"COVID-19"[Supplementary Concept] OR "SARS-CoV-2"[Supplementary Concept]) **AND** ("Disabled Persons"[Mesh] OR "Health Services for Persons with Disabilities"[Mesh] OR "Para-Athletes"[Mesh] OR "Disability Evaluation"[Mesh] OR "Disability Studies"[Mesh] OR "Learning Disabilities"[Mesh] OR "Intellectual Disability"[Mesh] OR "Developmental Disabilities"[Mesh] OR disab*[tw] OR impair*[tw] OR "special needs"[tw] OR "special education"[tw])

Web of Science – Core Collection:

(ALL=(vulnerab*) OR ALL=(refugee*) OR ALL=(asyl*) OR ALL=(justice) OR ALL=(injustice) OR ALL=(emmigr*) OR ALL=(immigr*) OR ALL=(migrant*) OR ALL=(migrat*) OR ALL=(ethnic) OR ALL=(disadvantaged) OR ALL=(prisoner*) OR ALL=(enslaved) OR ALL=(homebound) OR ALL=(homeless) OR ALL=(victim*) OR ALL=(violence) OR TS=(gender) OR ALL=(religion) OR ALL=(uninsured) OR ALL=(underinsured) OR ALL=(underserved) OR ALL=(equity) OR ALL=(poverty) OR ALL=(low income) OR ALL=(employment) OR ALL=(unemployment) OR ALL=(race) OR ALL=(indigenous) OR ALL=(institutionalized) OR ALL=(frail elderly) OR ALL=(comorbid*) OR ALL=(long-term care) OR ALL=(rural) OR ALL=(remote)) **AND** (ALL=(COVID-19) OR ALL=(SARS-CoV-2)) **AND** (ALL=(disab*) OR ALL=(impair*) OR ALL=(special needs) OR ALL=(special education))

CINAHL – through EBSCO host

(vulnerable or vulnerability or vulnerable populations or refugee* or asyl* or justice or injustice or immigrat* or emmigrat* or migrat* or migrant or ethnic or disadvantaged or

prisoner* or enslaved or homebound or homeless or victim* or violence or gender or religion or uninsured or underinsured or underserved or unemployment or employment or race or indigenous or institutionalized or long-term care or frail elderly or comorbid*or rural or remote) **AND** (covid-19 or 2019-ncov) **AND** (disability or disabilities or disabled or impairment or impaired or special needs or special education)

- Apply related words ☑
- Also search within the full text of the articles ☑
- Apply equivalent subjects ☑

APA PsycInfo – through EBSCO host

(vulnerable or vulnerability or vulnerable populations or refugee* or asyl* or justice or injustice or immigrat* or emmigrat* or migrat* or migrant or ethnic or disadvantaged or prisoner* or enslaved or homebound or homeless or victim* or violence or gender or religion or uninsured or underinsured or underserved or unemployment or employment or race or indigenous or institutionalized or long-term care or frail elderly or comorbid* or rural or remote) **AND** (covid-19 or 2019-ncov) **AND** (disability or disabilities or disabled or impairment or impaired or special needs or special education)

- Apply related words ☑

- Also search within the full text of the articles ☑
- Apply equivalent subjects ☑

AgeLine – through EBSCO host

(vulnerable or vulnerability or vulnerable populations or refugee* or asyl* or justice or injustice or immigrat* or emmigrat* or migrat* or migrant or ethnic or disadvantaged or prisoner* or enslaved or homebound or homeless or victim* or violence or gender or religion or uninsured or underinsured or underserved or unemployment or employment or race or indigenous or institutionalized or long-term care or frail or older or comorbid*or rural or remote) **AND** (covid-19 or 2019-ncov) **AND** (disability or disabilities or disabled or impairment or impaired or special needs or special education)

- Apply related words ☑
- Apply equivalent subjects ☑

ERIC: Educational Resource Information Center – through ProQuest

((vulnerable) OR (vulnerability) OR (refugee*) OR (asyl*) OR (justice) OR (injustice) or (immigrat*) or (emmigrat*) or (migrat*) or (migrant) or (ethnic) or (disadvantaged) or (prisoner*) or (enslaved) or (homebound) or (homeless) or (victim*) or (violence) or (gender) or (religion) or (uninsured) or (underinsured) or (underserved) or (unemployment) or (employment) or (race) or (indigenous) or (institutionalized) or (long-term care) or (frail elderly) or (comorbid*) or (rural) or (remote)) **AND** ((covid-19) or (2019-ncov)) **AND**

((disability) or (disabilities) or (disabled) or (impairment) or (impaired) or (special needs) or (special education))

Scopus

(ALL (vulnerab*) OR ALL (refugee*) OR ALL (asyl*) OR ALL (justice) OR ALL (injustice) OR ALL (emmigr*) OR ALL (immigr*) OR ALL (migrant*) OR ALL (migrat*) OR ALL (ethnic) OR ALL (disadvantaged) OR ALL (prisoner*) OR ALL (enslaved) OR ALL (homebound) OR ALL (homeless) OR ALL (victim*) OR ALL (violence) OR ALL (gender AND (role OR identity)) OR ALL (religion) OR ALL (uninsured) OR ALL (underinsured) OR ALL (underserved) OR ALL (equity) OR ALL (poverty) OR ALL (low AND income) OR ALL (employment) OR ALL (unemployment) ALL (race) OR ALL (indigenous) OR ALL (institutionalized) OR ALL (frail AND elderly) OR ALL (long-term AND care) OR ALL (rural) OR ALL (remote)) **AND** (ALL (covid-19) OR ALL (sars-cov-2)) **AND** (ALL (disab*) OR ALL (impair*) OR ALL (special AND needs) OR ALL (special AND education))

<u>Include:</u> Medicine; Social Sciences; Psychology; Nursing; Environmental Science; Neuroscience; Arts and Humanities; Computer Science; Business, Management, and Accounting; Health Professions; Economics, Econometrics and Finance; Dentistry; Decision Sciences.

MedRvix

(covid-19 or 2019-ncov) **AND** (disability or disabilities or disabled or impairment or impaired or special needs)

Note: the number of terms which can be included is limited.

SocARXIV

(covid-19 or 2019-ncov) **AND** (disability or disabilities or disabled or impairment or impaired or special needs)

PsyArXiv

(covid-19 or 2019-ncov) **AND** (disability or disabilities or disabled or impairment or impaired or special needs)
