# Supplementary material for: Health Risks and Consequences of a COVID-19 Infection for People with Disabilities: Scoping Review and Descriptive Thematic Analysis
Source: Int J Environ Res Public Health. 2021 Apr 20;18(8):4348. doi: 10.3390/ijerph18084348 (PMC8074171; doi:10.3390/ijerph18084348)
Supplement: Supplementary file 1 [file ijerph-18-04348-s001.zip › SM S3 - List of papers included.pdf]

Supplementary Material S3

List of 58 papers included (authors' alphabetic order)

1. Abedi, V., Olulana, O., Avula, V., Chaudhary, D., Khan, A., Shahjouei, S., Li, J., & Zand, R. (2020). Racial, Economic, and Health Inequality and COVID-19 Infection in the United States. *J Racial Ethn Health Disparities*, 1-11. https://doi.org/10.1007/s40615-020-00833-4
2. Alexander, R., Ravi, A., Barclay, H., Sawhney, I., Chester, V., Malcolm, V., Brolly, K., Mukherji, K., Zia, A., Tharian, R., Howell, A., Lane, T., Cooper, V., & Langdon, P. E. (2020). Guidance for the Treatment and Management of COVID-19 Among People with Intellectual Disabilities. *J Policy Pract Intellect Disabil*. https://doi.org/10.1111/jppi.12352
3. Andrews, E. E., Ayers, K. B., Brown, K. S., Dunn, D. S., & Pilarski, C. R. (2020). No body is expendable: Medical rationing and disability justice during the COVID-19 pandemic. *Am Psychol*. https://doi.org/10.1037/amp0000709
4. Armitage, R., & Nellums, L. B. (2020). The COVID-19 response must be disability inclusive. *Lancet Public Health*, *5*(5), e257. https://doi.org/10.1016/s2468-2667(20)30076-1
5. Azarpazhooh, M. R., Amiri, A., Morovatdar, N., Steinwender, S., Rezaei Ardani, A., Yassi, N., Biller, J., Stranges, S., Tokazebani Belasi, M., Neya, S. K., Khorram, B., Sheikh Andalibi, M. S., Arsang-Jang, S., Mokhber, N., & Di Napoli, M. (2020). Correlations between COVID-19 and burden of dementia: An ecological study and review of literature. *J Neurol Sci*, *416*, 117013. https://doi.org/10.1016/j.jns.2020.117013
6. Balestrini, S., Koepp, M. J., Gandhi, S., Rickman, H., Shin, G. Y., Houlihan, C., Anders-Cannon, J., Silvennoinen, K., Xiao, F., Zagaglia, S., Hudgell, K., Ziomek, M., Haimes, P., Sampson, A., Parker, A., Cross, J. H., Pardington, R., Nastouli, E., Swanton, C., Sander, J. W., & Sisodiya, S. (2020). Clinical outcomes of SARS-CoV-2 pandemic in long-term care facilities for people with epilepsy: observational study. *medRxiv*, 2020.2006.2010.20123281. https://doi.org/10.1101/2020.06.10.20123281
7. Ben-Pazi, H., Beni-Adani, L., & Lamdan, R. (2020). Accelerating Telemedicine for Cerebral Palsy During the COVID-19 Pandemic and Beyond. *Front Neurol*, *11*, 746. https://doi.org/10.3389/fneur.2020.00746
8. Boyle, C. A., Fox, M. H., Havercamp, S. M., & Zubler, J. (2020). The public health response to the COVID-19 pandemic for people with disabilities. *Disabil Health J*, *13*(3), 100943. https://doi.org/10.1016/j.dhjo.2020.100943
9. Brown, E. E., Kumar, S., Rajji, T. K., Pollock, B. G., & Mulsant, B. H. (2020). Anticipating and Mitigating the Impact of the COVID-19 Pandemic on Alzheimer's Disease and Related Dementias [Article]. *American Journal of Geriatric Psychiatry*, *28*(7), 712-721. https://doi.org/10.1016/j.jagp.2020.04.010
10. Chase, J. (2020). Caring for Frail Older Adults During COVID-19: Integrating Public Health Ethics into Clinical Practice. *J Am Geriatr Soc*, *68*(8), 1666-1670. https://doi.org/10.1111/jgs.16666
11. Chen, B., & McNamara, D. M. (2020). Disability Discrimination, Medical Rationing and COVID-19. *Asian Bioeth Rev*, 1-8. https://doi.org/10.1007/s41649-020-00147-x
12. Courtenay, K., & Perera, B. (2020). COVID-19 and people with intellectual disability: impacts of a pandemic. *Ir J Psychol Med*, *37*(3), 231-236. https://doi.org/10.1017/ipm.2020.45
13. Cox, D. J., Plavnick, J. B., & Brodhead, M. T. (2020). A Proposed Process for Risk Mitigation During the COVID-19 Pandemic. *Behav Anal Pract*, *13*(2), 1-7. https://doi.org/10.1007/s40617-020-00430-1

14. De Cauwer, H., & Spaepen, A. (2020). Are patients with Down syndrome vulnerable to life-threatening COVID-19? *Acta Neurol Belg*, 1-3. https://doi.org/10.1007/s13760-020-01373-8

15. Devita, M., Bordignon, A., Sergi, G., & Coin, A. The psychological and cognitive impact of Covid-19 on individuals with neurocognitive impairments: research topics and remote intervention proposals. *Aging Clinical and Experimental Research*. https://doi.org/10.1007/s40520-020-01637-6
16. Eskytė, I., Lawson, A., Orchard, M., & Andrews, E. (2020). Out on the Streets - Crisis, Opportunity and Disabled People in the Era of Covid-19: Reflections from the UK. *Alter*. https://doi.org/10.1016/j.alter.2020.07.004
17. Favalli, E. G., Ingegnoli, F., De Lucia, O., Cincinelli, G., Cimaz, R., & Caporali, R. (2020). COVID-19 infection and rheumatoid arthritis: Faraway, so close! *Autoimmun Rev*, *19*(5), 102523. https://doi.org/10.1016/j.autrev.2020.102523
18. Fernández-Díaz, E., Iglesias-Sánchez, P. P., & Jambrino-Maldonado, C. (2020). Exploring WHO Communication during the COVID 19 Pandemic through the WHO Website Based on W3C Guidelines: Accessible for All? *Int J Environ Res Public Health*, *17*(16). https://doi.org/10.3390/ijerph17165663
19. Glover, R. E., van Schalkwyk, M. C. I., Akl, E. A., Kristjannson, E., Lotfi, T., Petkovic, J., Petticrew, M. P., Pottie, K., Tugwell, P., & Welch, V. (2020). A framework for identifying and mitigating the equity harms of COVID-19 policy interventions. *J Clin Epidemiol*, *128*, 35-48. https://doi.org/10.1016/j.jclinepi.2020.06.004
20. Goggin, G., & Ellis, K. (2020). Disability, communication, and life itself in the COVID-19 pandemic. *Health Sociology Review*, *29*(2), 168-176. https://doi.org/10.1080/14461242.2020.1784020
21. Guidry-Grimes, L., Savin, K., Stramondo, J. A., Reynolds, J. M., Tsaplina, M., Burke, T. B., Ballantyne, A., Kittay, E. F., Stahl, D., Scully, J. L., Garland-Thomson, R., Tarzian, A., Dorfman, D., & Fins, J. J. (2020). Disability Rights as a Necessary Framework for Crisis Standards of Care and the Future of Health Care. *Hastings Cent Rep*, *50*(3), 28-32. https://doi.org/10.1002/hast.1128
22. Iaboni, A., Cockburn, A., Marcil, M., Rodrigues, K., Marshall, C., Garcia, M. A., Quirt, H., Reynolds, K. B., Keren, R., & Flint, A. J. (2020). Achieving Safe, Effective, and Compassionate Quarantine or Isolation of Older Adults With Dementia in Nursing Homes. *Am J Geriatr Psychiatry*, *28*(8), 835-838. https://doi.org/10.1016/j.jagp.2020.04.025
23. Ibanez, A., & Kosik, K. S. (2020). COVID-19 in older people with cognitive impairment in Latin America. *Lancet Neurol*, *19*(9), 719-721. https://doi.org/10.1016/s1474-4422(20)30270-2

24. Jesus, T. S., Landry, M. D., & Jacobs, K. (2020). A 'new normal' following COVID-19 and the economic crisis: Using systems thinking to identify challenges and opportunities in disability, telework, and rehabilitation. *Work*, *67*(1), 37-46. https://doi.org/10.3233/wor-203250
25. Korupolu, R., Stampas, A., Gibbons, C., Hernandez Jimenez, I., Skelton, F., & Verduzco-Gutierrez, M. (2020). COVID-19: Screening and triage challenges in people with disability due to Spinal Cord Injury. *Spinal Cord Ser Cases*, *6*(1), 35. https://doi.org/10.1038/s41394-020-0284-7
26. Kuper, H., Banks, L. M., Bright, T., Davey, C., & Shakespeare, T. (2020). Disability-inclusive COVID-19 response: What it is, why it is important and what we can learn from the United Kingdom's response. *Wellcome Open Res*, *5*, 79. https://doi.org/10.12688/wellcomeopenres.15833.1
27. Landes, S. D., Turk, M. A., Formica, M. K., McDonald, K. E., & Stevens, J. D. (2020). COVID-19 outcomes among people with intellectual and developmental disability living in residential group homes in New York State. *Disabil Health J*, *13*(4), 100969. https://doi.org/10.1016/j.dhjo.2020.100969

28. Landi, D., Ponzano, M., Nicoletti, C. G., Cecchi, G., Cola, G., Mataluni, G., Mercuri, N. B., Sormani, M. P., & Marfia, G. A. (2020). Adherence to social distancing and use of personal protective equipment and the risk of SARS-CoV-2 infection in a cohort of patients with multiple sclerosis. *Mult Scler Relat Disord*, *45*, 102359. https://doi.org/10.1016/j.msard.2020.102359
29. Llanes Mabalot, C. (2020). Advocacy During Covid-19. *Exceptional Parent*, *50*(6), 14-16. http://search.ebscohost.com/login.aspx?direct=true&db=cin20&AN=143897146&site=ehost-live&scope=site
30. Lund, E. M. (2020). Interpersonal violence against people with disabilities: Additional concerns and considerations in the COVID-19 pandemic. *Rehabil Psychol*, *65*(3), 199-205. https://doi.org/10.1037/rep0000347
31. Lund, E. M., & Ayers, K. B. (2020). Raising awareness of disabled lives and health care rationing during the COVID-19 pandemic. *Psychol Trauma*, *12*(S1), S210-s211. https://doi.org/10.1037/tra0000673
32. Manchanda, E. C. C., Sanky, C., & Appel, J. M. Crisis Standards of Care in the USA: A Systematic Review and Implications for Equity Amidst COVID-19. *J Racial Ethn Health Disparities*. https://doi.org/10.1007/s40615-020-00840-5
33. Manto, M., Dupre, N., Hadjivassiliou, M., Louis, E. D., Mitoma, H., Molinari, M., Shaikh, A. G., Soong, B. W., Strupp, M., Van Overwalle, F., & Schmahmann, J. D. (2020). Medical and Paramedical Care of Patients With Cerebellar Ataxia During the COVID-19 Outbreak: Seven Practical Recommendations of the COVID 19 Cerebellum Task Force. *Front Neurol*, *11*, 516. https://doi.org/10.3389/fneur.2020.00516
34. McKinney, E. L., McKinney, V., & Swartz, L. (2020). COVID-19, disability and the context of healthcare triage in South Africa: Notes in a time of pandemic. *African Journal of Disability*, *9*, Article a766. https://doi.org/10.4102/ajod.v9i0.766
35. Mello MM, Persad G, White DB. Respecting Disability Rights — Toward Improved Crisis Standards of Care. *New England Journal of Medicine* 2020;383(5):e26. doi: 10.1056/NEJMp2011997
36. Muruganandam, P., Neelamegam, S., Menon, V., Alexander, J., & Chaturvedi, S. K. (2020). COVID-19 and Severe Mental Illness: Impact on patients and its relation with their awareness about COVID-19. *Psychiatry Res*, *291*, 113265. https://doi.org/10.1016/j.psychres.2020.113265
37. Okonkwo, N. E., Aguwa, U. T., Jang, M., Barré, I. A., Page, K. R., Sullivan, P. S., Beyrer, C., & Baral, S. (2020). COVID-19 and the US response: accelerating health inequities. *BMJ Evid Based Med*. https://doi.org/10.1136/bmjebm-2020-111426
38. Olulana, O., Abedi, V., Avula, V., Chaudhary, D., Khan, A., Shahjouei, S., Li, J., & Zand, R. (2020). Regional Association of Disability and SARS-CoV-2 Infection in 369 Counties of the United States. *medRxiv*. https://doi.org/10.1101/2020.06.24.20139212
39. Palipana, D. (2020). COVID-19 and spinal cord injuries: The viewpoint from an emergency department resident with quadriplegia [Article]. *EMA - Emergency Medicine Australasia*, *32*(4), 692-693. https://doi.org/10.1111/1742-6723.13525
40. Parsons, J. A., & Johal, H. K. (2020). Best interests versus resource allocation: could COVID-19 cloud decision-making for the cognitively impaired? *J Med Ethics*, *46*(7), 447-450. https://doi.org/10.1136/medethics-2020-106323
41. Pineda, V. S., & Corburn, J. (2020). Disability, Urban Health Equity, and the Coronavirus Pandemic: Promoting Cities for All. *J Urban Health*, *97*(3), 336-341. https://doi.org/10.1007/s11524-020-00437-7
42. Sabatello, M., Burke, T. B., McDonald, K. E., & Appelbaum, P. S. (2020). Disability, Ethics, and Health Care in the COVID-19 Pandemic. *Am J Public Health*, *110*(10), 1523-1527. https://doi.org/10.2105/ajph.2020.305837

43. Sabatello, M., Landes, S. D., & McDonald, K. E. (2020). People With Disabilities in COVID-19: Fixing Our Priorities. *American Journal of Bioethics*, *20*(7), 187-190. https://doi.org/10.1080/15265161.2020.1779396
44. Safta-Zecheria, L. (2020). Challenges posed by COVID-19 to the health of people with disabilities living in residential care facilities in Romania. *Disability & Society*, *35*(5), 837-843. https://doi.org/10.1080/09687599.2020.1754766
45. Sakellariou, D., Malfitano, A. P. S., & Rotarou, E. S. (2020). Disability inclusiveness of government responses to COVID-19 in South America: a framework analysis study. *International Journal for Equity in Health*, *19*(1), Article 131. https://doi.org/10.1186/s12939-020-01244-x
46. Scully, J. L. (2020). Disability, Disablism, and COVID-19 Pandemic Triage. *J Bioeth Inq*, 1-5. https://doi.org/10.1007/s11673-020-10005-y
47. Senjam, S. S. (2020). Impact of COVID-19 pandemic on people living with visual disability. *Indian J Ophthalmol*, *68*(7), 1367-1370. https://doi.org/10.4103/ijo.IJO_1513_20
48. Sholas, M. G. (2020). The actual and potential impact of the novel 2019 coronavirus on pediatric rehabilitation: A commentary and review of its effects and potential disparate influence on Black, Latinx and Native American marginalized populations in the United States. *J Pediatr Rehabil Med*. https://doi.org/10.3233/prm-200722
49. Singh, S. (2020). Disability ethics in the coronavirus crisis. *J Family Med Prim Care*, *9*(5), 2167-2171. https://doi.org/10.4103/jfmpc.jfmpc_588_20
50. Solomon MZ, Wynia MK, Gostin LO. Covid-19 Crisis Triage — Optimizing Health Outcomes and Disability Rights. *New England Journal of Medicine* 2020;383(5):e27. doi: 10.1056/NEJMp2008300
51. Stillman, M. D., Capron, M., Alexander, M., Di Giusto, M. L., & Scivoletto, G. (2020). COVID-19 and spinal cord injury and disease: results of an international survey. *Spinal Cord Ser Cases*, *6*(1), 21. https://doi.org/10.1038/s41394-020-0275-8
52. Tohidast, S. A., Mansuri, B., Bagheri, R., & Azimi, H. (2020). Provision of speech-language pathology services for the treatment of speech and language disorders in children during the COVID-19 pandemic: Problems, concerns, and solutions [Article]. *International Journal of Pediatric Otorhinolaryngology*, *138*, Article 110262. https://doi.org/10.1016/j.ijporl.2020.110262
53. Turk, M. A., Landes, S. D., Formica, M. K., & Goss, K. D. (2020). Intellectual and developmental disability and COVID-19 case-fatality trends: TriNetX analysis. *Disabil Health J*, *13*(3), 100942. https://doi.org/10.1016/j.dhjo.2020.100942
54. Turk, M. A., & McDermott, S. (2020). The COVID-19 pandemic and people with disability [Editorial]. *Disabil Health J*, *13*(3), Article 100944. https://doi.org/10.1016/j.dhjo.2020.100944
55. Umucu, E., & Lee, B. (2020). Examining the impact of COVID-19 on stress and coping strategies in individuals with disabilities and chronic conditions. *Rehabil Psychol*, *65*(3), 193-198. https://doi.org/10.1037/rep0000328
56. Waldman, H. B., Rader, R., Keller, S. M., & Perlman, S. P. (2020). Who's Next? *Exceptional Parent*, *50*(5), 16-18. http://search.ebscohost.com/login.aspx?direct=true&db=cin20&AN=143154671&site=ehost-live&scope=site
57. Waldman, H. B., Rader, R., & Perlman, S. P. (2020). What Are We Learning? *Exceptional Parent*, *50*(6), 17-19. http://search.ebscohost.com/login.aspx?direct=true&db=cin20&AN=143897147&site=ehost-live&scope=site
58. Yap, J., Chaudhry, V., Jha, C. K., Mani, S., & Mitra, S. (2020). Are responses to the pandemic inclusive? A rapid virtual audit of COVID-19 press briefings in LMICs. *World Dev*, *136*, 105122. https://doi.org/10.1016/j.worlddev.2020.105122
